# Heel-to-toe drop effects on biomechanical and muscle synergy responses during uphill walking

**DOI:** 10.3389/fbioe.2024.1385264

**Published:** 2024-05-10

**Authors:** Liang Jiang, Feng Qu, Zihan Yang, Xue Chen, Xianzhi Gao, Qing Sun, Bo Huo

**Affiliations:** ^1^ School of Aerospace Engineering, Beijing Institute of Technology, Beijing, China; ^2^ Biomechanics Laboratory, Department of Kinesiology, Beijing Sport University, Beijing, China; ^3^ Fashion Accessory Art and Engineering College, Beijing Institute of Fashion Technology, Beijing, China; ^4^ Sport Biomechanics Center, Institute of Artificial Intelligence in Sports, Capital University of Physical Education and Sports, Beijing, China

**Keywords:** slope, heel-to-toe drop, gait, joint work, frontal plane, muscle synergy

## Abstract

Uphill walking is a common task encountered in daily life, with steeper inclines potentially imposing greater biomechanical and neuromuscular demands on the human body. The heel-to-toe drop (HTD) in footwear may influence the biomechanical and neuromuscular pattern of uphill walking; but the impact remains unclear. Adjustments in HTD can modulate biomechanical and neuromuscular patterns, mitigating the demands and optimizing the body’s response to different inclinations. We hypothesize that adjustments in HTD can modulate biomechanical and neuromuscular patterns, mitigating the demands and optimizing the body’s response to different inclinations. Nineteen healthy men walked on an adjustable slope walkway, with varied inclinations (6°, 12°, 20°) and HTD shoes (10mm, 25mm, 40 mm), while the marker positions, ground reaction forces and electromyography data were collected. Our study reveals that gait temporo-spatial parameters are predominantly affected by inclination over HTD. Inclination has a more pronounced effect on kinematic variables, while both inclination and HTD significantly modulate kinetic and muscle synergy parameters. This study demonstrates that an increase in the inclination leads to changes in biomechanical and neuromuscular responses during uphill walking and the adjustment of HTD can modulate these responses during uphill walking. However, the present study suggests that an increased HTD may lead to elevated loads on the knee joint and these adverse effects need more attention.

## 1 Introduction

Slopes are commonly encountered during hiking and everyday activities. Navigating slopes presents more of a challenge than flat terrain. Ascending a slope increases metabolic work (Minetti et al., 2002; Franz and Kram, 2012; Yang et al., 2019) and necessitates adjustments in the activity of the upper and lower limbs and trunk muscles to maintain balance while progressing both forward and upward (Leroux et al., 2002; Lay et al., 2006; Kimel-Naor et al., 2017). The increased incline alters gait patterns and demands greater physiological function from joints and muscles, leading to reduced step length, speed, and stride frequency (Kimel-Naor et al., 2017), as well as increased positive work in the ankle, knee, and hip joints (Alexander et al., 2017; Yang et al., 2019), and heightened lower limb muscle activation (Lay et al., 2007). The likelihood of falls is greater when walking on slopes compared to level surfaces, particularly for older adults and individuals with disabilities (Kannus et al., 1999; Miller et al., 2001; Redfern et al., 2001). Epidemiological studies have also indicated a higher incidence of lower limb injuries, such as skin abrasions, blisters, muscle strains, fractures, and ankle sprains, during mountain climbing and hiking (Heggie and Heggie, 2004; Johnson et al., 2007). Consequently, investigating safer sports strategies for slope activities is of great importance.

Shoes directly contact with the ground and their structure and materials affect human motion (Wiedemeijer and Otten, 2018; Sun et al., 2020). The heel-to-toe drop (HTD) of shoes, which refers to the difference in thickness between the forefoot and heel parts of the sole, has been identified as a factor that may influence biomechanical parameters during walking (Cowley et al., 2009; Cronin, 2014). As HTD increases, several changes of gait characteristics have been observed. The gait cycle time tends to increase, while gait speed slows down due to a prolonged support period and shortened swing period (Barkema et al., 2012; Di Sipio et al., 2018). The range of motion (RoM) of the ankle, knee or hip decreases during level walking when wearing high-heel shoes (Mika et al., 2012; Annoni et al., 2014). Additionally, an increased HTD during level walking may induce alterations in kinetic and electromyographic parameters of the lower limb (Simonsen et al., 2012). These alterations are commonly associated with an elevated risk of injury to the ankle and knee joints (Barkema et al., 2012; Mika et al., 2012; Barnish and Barnish, 2016). However, the aforementioned results are based on level walking, and research on the adjustment of lower limb biomechanical patterns in response to HTD during slope walking is still limited.

Walking is a physical activity that requires a high degree of coordination between joints and muscles to be completed (Bianchi et al., 1998; Lacquaniti et al., 2012; Esmaeili et al., 2022). To simplify the high degree of freedom in the human motor system, muscle activity can be divided into the groups with fixed spatial structures that are activated together, known as muscle synergy or motor modules (Cappellini et al., 2006; Ivanenko et al., 2006; Bizzi and Cheung, 2013). Pathological conditions, such as stroke and cerebral palsy, can influence this coordination (Clark et al., 2010; Steele et al., 2015). When walking uphill, the number of muscle synergy patterns remains the same as level walking (Rozumalski et al., 2017; Saito et al., 2018; Liu and Gutierrez-Farewik, 2023). However, the frequencies of respective synergies vary due to changes in mechanical demands between uphill and level walking (Janshen et al., 2017), where uphill walking requires the lower limb muscles to work more to lift up and maintain balance (Wall-Scheffler et al., 2010; Franz and Kram, 2012). Furthermore, HTD influences the function of lower limb muscles while walking (Park et al., 2010; Simonsen et al., 2012), and studies on the impact of HTD on muscle synergy and muscle work during uphill walking are relatively scarce.

Due to the differences of biomechanically and muscle functional roles between level and slope gait (Pickle et al., 2016; Wen et al., 2019), the findings from level walking may not be directly applicable to slope walking. Existing patents have introduced footwear with adjustable heel heights (Kumar et al., 2020). The underlying principle of these patents involves modulating heel height to align the foot in a more natural orientation relative to the inclination of the surface. Heel elevation during uphill walking may reduce dorsiflexion angle, simulating a flat-foot position, but its impact on muscle coordination varies among individuals and is under-researched. The interaction between inclination and HTD on biomechanics and neuromuscular responses remains unclear.

We hypothesize that adjustments in HTD can modulate biomechanical and neuromuscular patterns, mitigating the demands and optimizing the body’s response to different inclinations.

## 2 Methods

### 2.1 Participants

Nineteen healthy men (Age: 23.4 ± 2.1 years; Height: 176.5 ± 5.3 cm; Weight: 70.4 ± 7.9 kg; Shoe size: 42 or 43 EU) free of any neurological or musculoskeletal disorders volunteered to participate in the study. Each subject signed an informed consent form approved by the Institutional Review Board of Capital University of Physical Education and Sports.

### 2.2 Conditions of inclination and HTD

**FIGURE 1:**
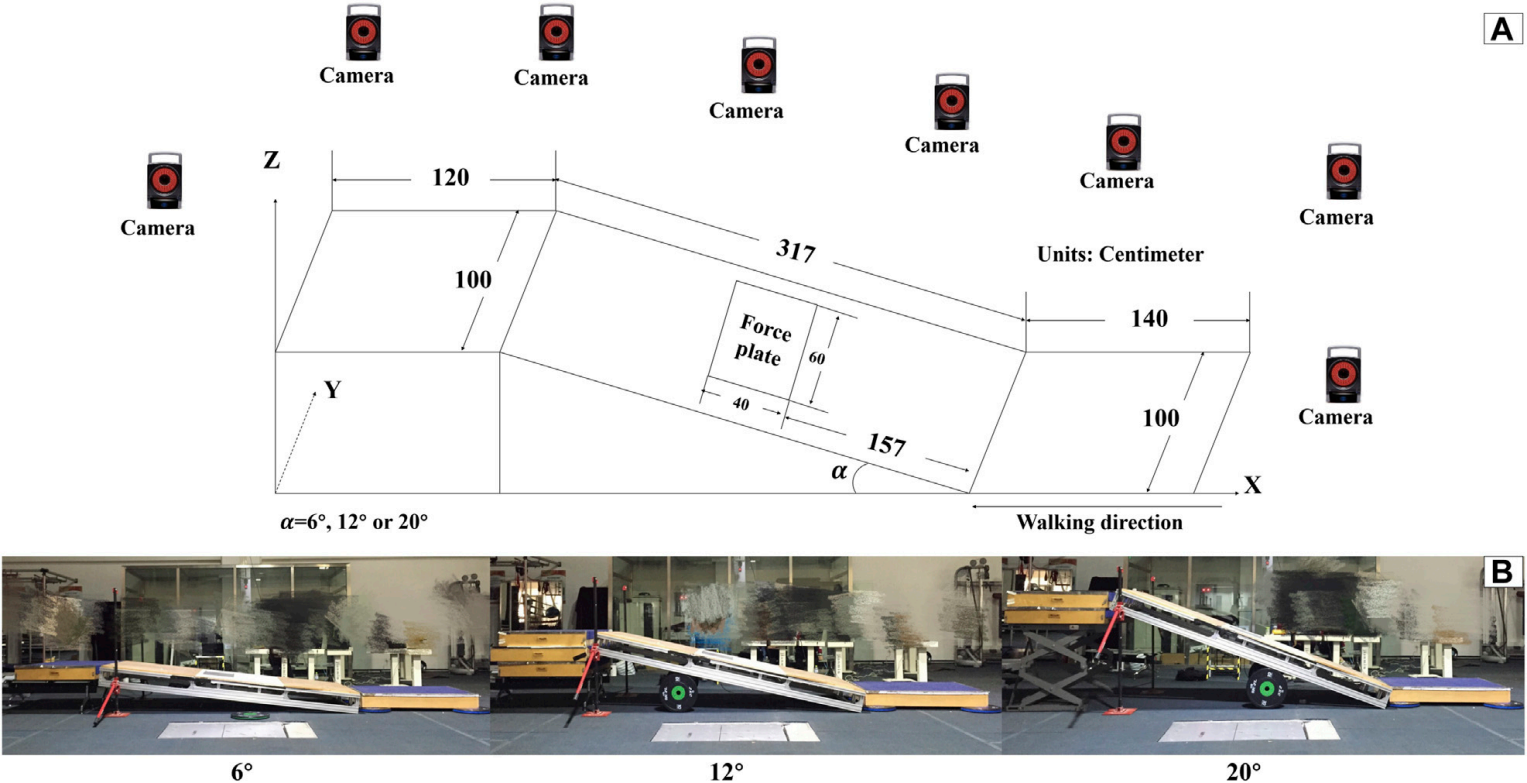
Adjustable slope walkway. **(A)** diagram. **(B)** Scene pictures.

**FIGURE 2:**
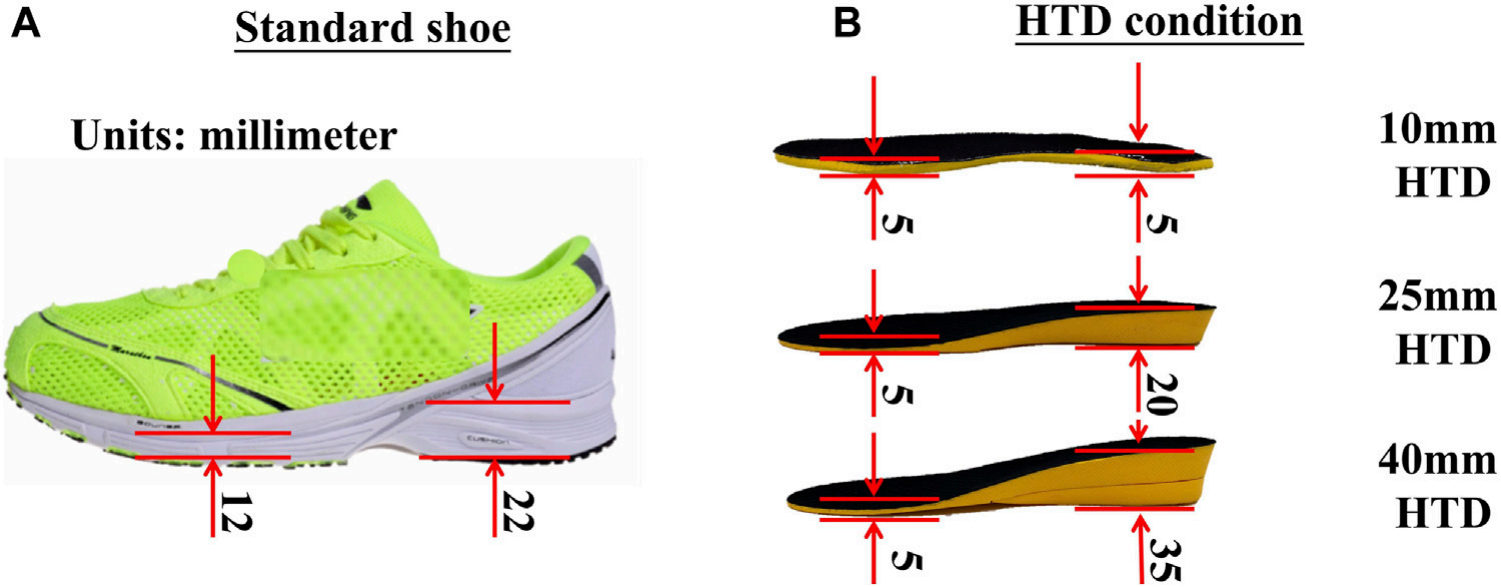
The thickness of sole and insole during experiments. **(A)** The photo of a standard shoe. **(B)** The HTD condition of an insole.

An adjustable slope walkway was built first and it was prepared according to the procedure used by previous research (Yang et al., 2019). The main structure of the slope walkway is an aluminum alloy frame and wood surface. The force plate (Kistler 9281CA, Switzerland) was mounted on a vertical strut in the middle of the slope walkway. Wooden flat walkways are located in front and at the end of the slope walkway, The front-end wooden walkway platform is 1.4 m in length and 1 m in width. The rear wooden walkway platform measures 1.2 m in length and 1 m in width. The sloped walkway extends for 3.17 m in length and is 1 m wide (Figure 1). The inclinations was adjusted to 6°, 12° or 20° (Earhart and Bastian, 2000; Prentice et al., 2004; Lay et al., 2006). After initially selecting the inclinations at random, the subjects wore standard shoes under various HTD conditions (10mm, 25mm, and 40 mm) randomly as shown in Figure 2. The remaining two slope conditions, each with three HTD settings, were conducted in a randomized sequence. Five practice trials and three uphill walking trails were completed for each inclination and HTD condition at subject’s self-paced speed. Trials were discarded if the participant’s right foot stepped on the force platform incompletely, or if the participant targeted the platform, to ensure movement authenticity and prevent unnatural gait patterns from biasing the results.

### 2.3 Motion capture, GRF and EMG measurements

Twenty-nine reflective markers were placed according to a modified Halen Hayes Marker set (Vaughan et al., 1999). Three reflective markers was placed on the three corners of the force platform to mark its position. Kinematic data were collected at 200 Hz with an eight-camera 3D Optical Capture system (Motion Analysis Raptor-4, United States). Ground reaction force (GRF) was recorded at 1000 Hz. Surface electromyography (EMG) data of the following eight muscles of the right lower limb were recorded at 2000 Hz using a wireless EMG system (Delsys Trigno, United States): tibialis anterior (TA), gastrocnemius medialis (GM), gastrocnemius lateralis (GL), rectus femoris (RF), vastus medialis (VM), vastus lateralis (VL), semitendinosus (ST), biceps femoris (long head, BF). Location of electrodes using the same method of one previous research (Hermens et al., 2000). Marker position, GRF and EMG data were synchronized using an external trigger signal.

### 2.4 Kinematic and kinetic analysis

Kinematic data was further processed with Cortex (version 2.6, Motion Analysis Corporation, Santa Rosa, CA). The software was then used to transform the GRFs of the force plate and align them with the global reference system. The processing of GRF data was carried out using Matlab programming (MathWorks, Natick, MA). Kinematic and GRF data were low pass filtered (4th-order, zero-lag, Butterworth), with a cut off frequency of 8 Hz and 15Hz, respectively (Yang et al., 2019). Heel-strikes (HS) and toe-offs (TO) of the right foot were identified according to the 10N-threshold vertical GRF or a foot marker-based algorithm (O'Connor et al., 2007). For each gait cycle, the temporo-spatial parameters were calculated such as gait speed, stride length, duration of stance phase, duration of double stance phase, and cadence. The joint kinematics coordinate reference systems were defined according to the recommendation of the International Society of Biomechanics (Wu and Cavanagh, 1995; Wu et al., 2002). The range of motion (RoM) and the joint angles at the moment of HS for the ankle, knee, and hip joints were assessed. Joint moment were calculated according to the procedure used by previous research (Winter 1980; Vaughan et al., 1999). Joint powers were calculated by multiplying joint angular velocity by joint moment (Winter 1991; Eng and Winter 1995). Joint moments and powers were normalized to body weight (BW).

### 2.5 Muscle synergy analysis

EMG activity was analyzed using R script (R v3.6.3, R Core TEAM, 2020, R Foundation for Statistical Computing, Vienna, Austria). The raw EMG data was band-pass filtered between 50 and 500Hz, then full-wave rectified, and finally low-pass filtered (4th-order, zero-lag, Butterworth) with a cut-off frequency of 20 Hz for to create a linear envelope (Santuz et al., 2020). EMG data of each muscle was normalized to its maximum value across all conditions (Devarajan and Cheung, 2014). Each gait cycle was then time-normalized to 200 points, with 100 points each assigned to support and swing phases (Santuz et al., 2018; Santuz et al., 2019). The classical Gaussian non-negative matrix factorization (NNMF) algorithm extracted muscle synergies, organized into a matrix V with dimensions m×n (m rows and n columns). Where n represents the number of normalized time points. The matrix V was factorized using NNMF, such that ${V\approx V}_{R}=\text{WH}$ , with the new matrix VR reconstructed by multiplying the two matrices W and H to approximate the original matrix V. The motor primitives matrix H contains time-dependent coefficients of the factorization with dimensions r×n , where the minimum number of rows r represents the number of synergies required to satisfactorily reconstruct the original set of signals V (Lacquaniti et al., 2012). Update rules for matrices W and H were applied, and reconstruction quality was measured by *R* ^2^, with convergence at a change ≤0.01% over 20 iterations (Santuz et al., 2017). The minimum synergies were determined by fitting *R* ^2^ values to synergies and recalculating errors (Cheung et al., 2005) after removing points until two remained or error was <10⁻⁴ (Santuz et al., 2019). Motor primitives were classified using K-means clustering, clustering based on the distance between features, and discarding irrelevant primitives by *R* ^2^ comparison (Santuz et al., 2020). The center of activity (CoA) and full width at half maximum (FWHM) were calculated for activation patterns under various conditions using polar coordinates and averaged for stance and swing phases (Cappellini et al., 2016).

### 2.6 Statistics

The values for the three trials were averaged for each subject at each HTD and inclination. A two-way repeated measures ANOVA was utilized to evaluate the influence of HTD and inclination on gait temporo-spatial parameters, kinematics, kinetics, and muscle synergies. Significant main or interaction effects were identified (*p* < 0.05). Post hoc analyses with the Tukey test ( α = 0.05) elucidated these effects. Effect sizes were quantified using Partial Eta Squared ( $\eta_{p}^{2}$ ), with values of 0.01, 0.06, and 0.14 representing small, moderate, and large effects, respectively. All statistical analyses were performed using SPSS v23 software (SPSS Inc., Chicago, IL, United States).

## 3 Results

### 3.1 Inclination rather than HTD influences the temporo-spatial parameters of gait

No inclination✕HTD interaction effects were observed for temporo-spatial parameters. A significant main effect of inclinations (Table 1) was observed for gait speed (F = 22.56, *p* < 0.001, $\eta_{p}^{2}$ = 0.85), stride length (F = 13.07, *p* < 0.001, $\eta_{p}^{2}$ = 0.71), stance duration (F = 30.96, *p* < 0.001, $\eta_{p}^{2}$ = 0.78), double stance (F = 14.03, *p* < 0.001, $\eta_{p}^{2}$ = 0.61), and cadence (F = 18.97, *p* < 0.001, $\eta_{p}^{2}$ = 0.83). There was no significant main effect observed for HTD in temporo-spatial parameters.

**TABLE 1:** Mean (SD) gait parameters used to describe walking on three inclinations with three HTD-levels.

Variable					ANOVA results									
	HTD				Interaction			Inclination			HTD			Post-hoc
	Inclination	10 mm	25 mm	40 mm	*F*	*p*	$\eta_{p}^{2}$	*F*	*p*	$\eta_{p}^{2}$	*F*	*p*	$\eta_{p}^{2}$	
Gait speed [m/s]	6°	2.34 (0.34)	2.23 (0.22)	2.22 (0.25)	.39	.81	.21	22.56	<.01	.85	1.86	.19	.17	6° > 12°, 6° > 20°, 12° > 20°
	12°	2.07 (0.3)	1.99 (0.13)	2.01 (0.16)										
	20°	1.74 (0.14)	1.75 (0.23)	1.74 (0.22)										
Stride length [cm]	6°	140.07 (14.99)	135.73 (11.28)	134.95 (12.86)	.51	.74	.25	13.07	<.01	.72	1.70	.14	.20	6° > 12°, 6° > 20°, 12° > 20°
	12°	130.17 (13.91)	126.47 (5.87)	126.85 (7.96)										
	20°	117.87 (8.26)	117.96 (10.16)	118.91 (9.84)										
Stance duration [%]	6°	63.01 (2.64)	63.57 (1.92)	63.34 (1.67)	.19	.33	.12	30.96	<.01	.78	1.13	.33	.11	6° < 12°, 6° < 20°, 12° < 20°
	12°	64.32 (1.66)	65.44 (1.08)	65.51 (0.82)										
	20°	67.14 (1.85)	66.61 (1.47)	67.23 (2.12)										
Double stance duration [%]	6°	13.54 (2.33)	14.01 (2.1)	13.57 (1.54)	2.37	.11	.21	14.03	<.01	.61	.14	.79	.02	6° < 20°, 12° < 20°
	12°	13.84 (1.48)	14.42 (1.3)	14.59 (1.26)										
	20°	17.26 (2.31)	15.9 (1.72)	16.01 (1.79)										
Cadence [steps/s]	6°	1.66 (0.11)	1.64 (0.12)	1.64 (0.09)	.39	.72	.04	18.97	<.01	.83	.37	.65	.04	6° > 12°, 6° > 20°, 12° > 20°
	12°	1.59 (0.12)	1.58 (0.09)	1.59 (0.09)										
	20°	1.48 (0.09)	1.48 (0.14)	1.46 (0.12)										

### 3.2 Inclination influences more kinematic parameters than HTD

**FIGURE 3:**
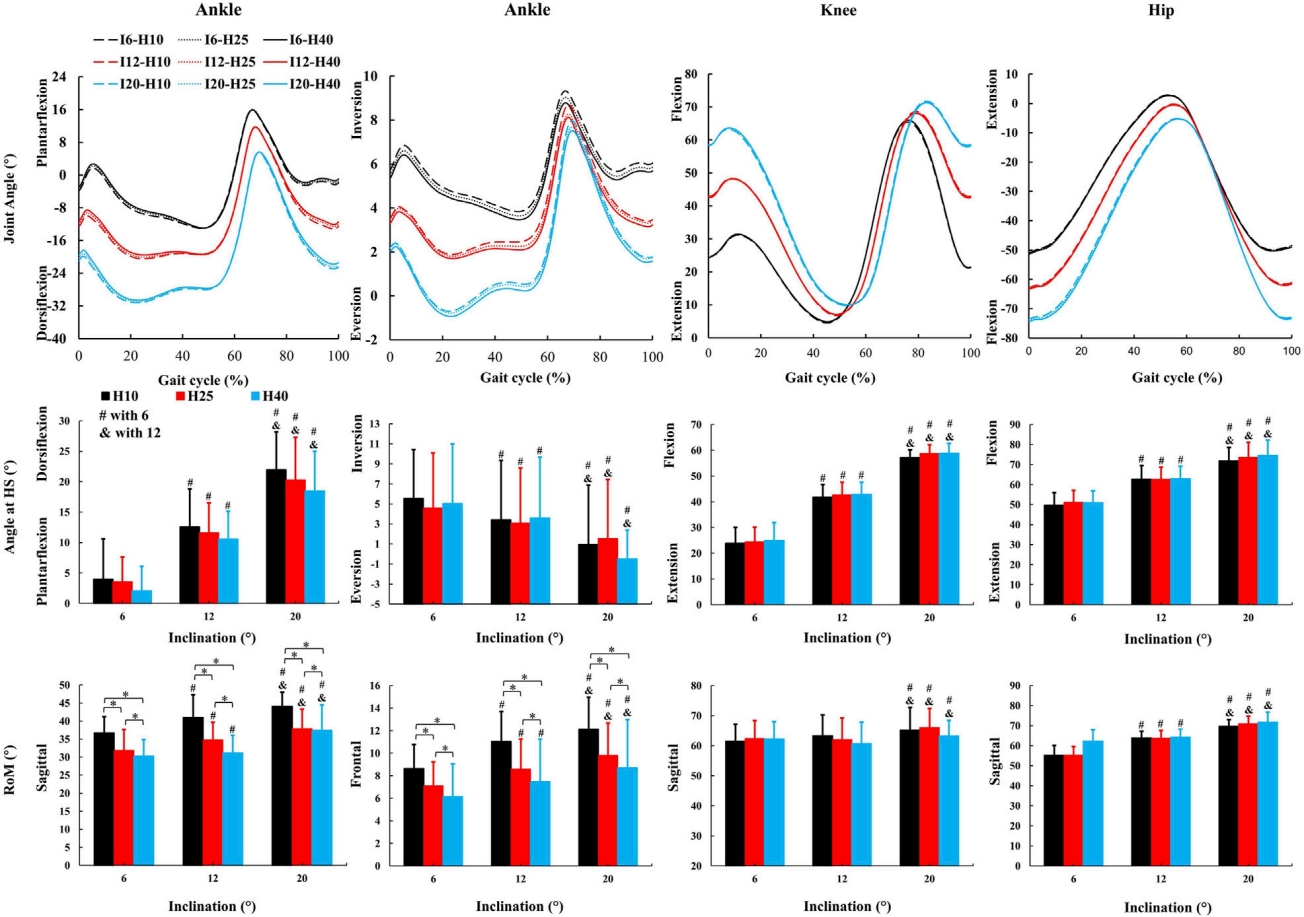
The average kinematic parameters (joint angle and characteristic values) during uphill walking with various inclinations and heel-to-toe drops (HS = Heel Strike, RoM = Range of Motion, I - inclination, H - HTD). *, #, & indicate a significant difference (*p* < 0.05).

No inclination ✕ HTD interaction effects were observed for kinematic parameters. A significant main effect of inclinations (Figure 3) was observed for ankle dorsiflexion angle at HS (F = 51.72, *p* < 0.001, $\eta_{p}^{2}$ = 0.93), ankle inversion angle at HS (F = 21.24, *p* < 0.001, $\eta_{p}^{2}$ = 0.84), knee flexion angle at HS (F = 417.39, *p* < 0.001, $\eta_{p}^{2}$ = 0.98), hip flexion angle at HS (F = 241.58, *p* < 0.001, $\eta_{p}^{2}$ = 0.98), ankle sagittal RoM (F = 18.93, *p* < 0.001, $\eta_{p}^{2}$ = 0.83), ankle frontal RoM (F = 30.91, *p* < 0.001, $\eta_{p}^{2}$ = 0.77), knee sagittal RoM (F = 5.68, *p* = 0.015, $\eta_{p}^{2}$ = 0.39), hip sagittal RoM (F = 172.52, *p* < 0.001, $\eta_{p}^{2}$ = 0.97). There was no significant main effect observed for HTD in kinematics parameters (Figure 3) except for ankle sagittal RoM (F = 33.71, *p* < 0.001, $\eta_{p}^{2}$ = 0.89) and ankle frontal RoM (F = 4.48, *p* = 0.049, $\eta_{p}^{2}$ = 0.53).

### 3.3 Both inclination and HTD modulate the kinetic parameters

**FIGURE 4:**
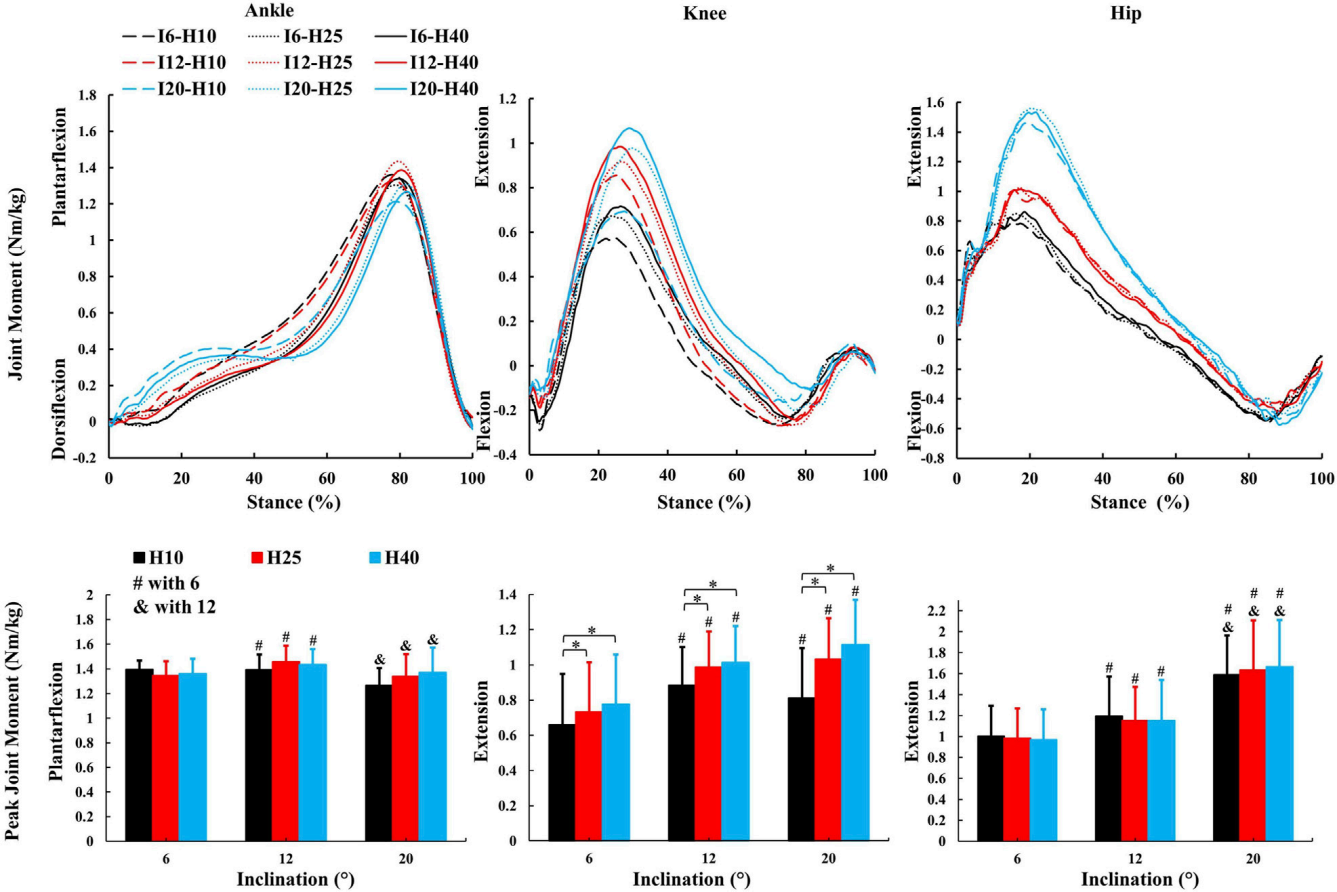
The average kinetic parameters (sagittal joint moment and peak value) during the stance phase of uphill walking with various inclinations and heel-to-toe drops (I - inclination, H - HTD). *, #, & indicate a significant difference (*p* < 0.05).

**FIGURE 5:**
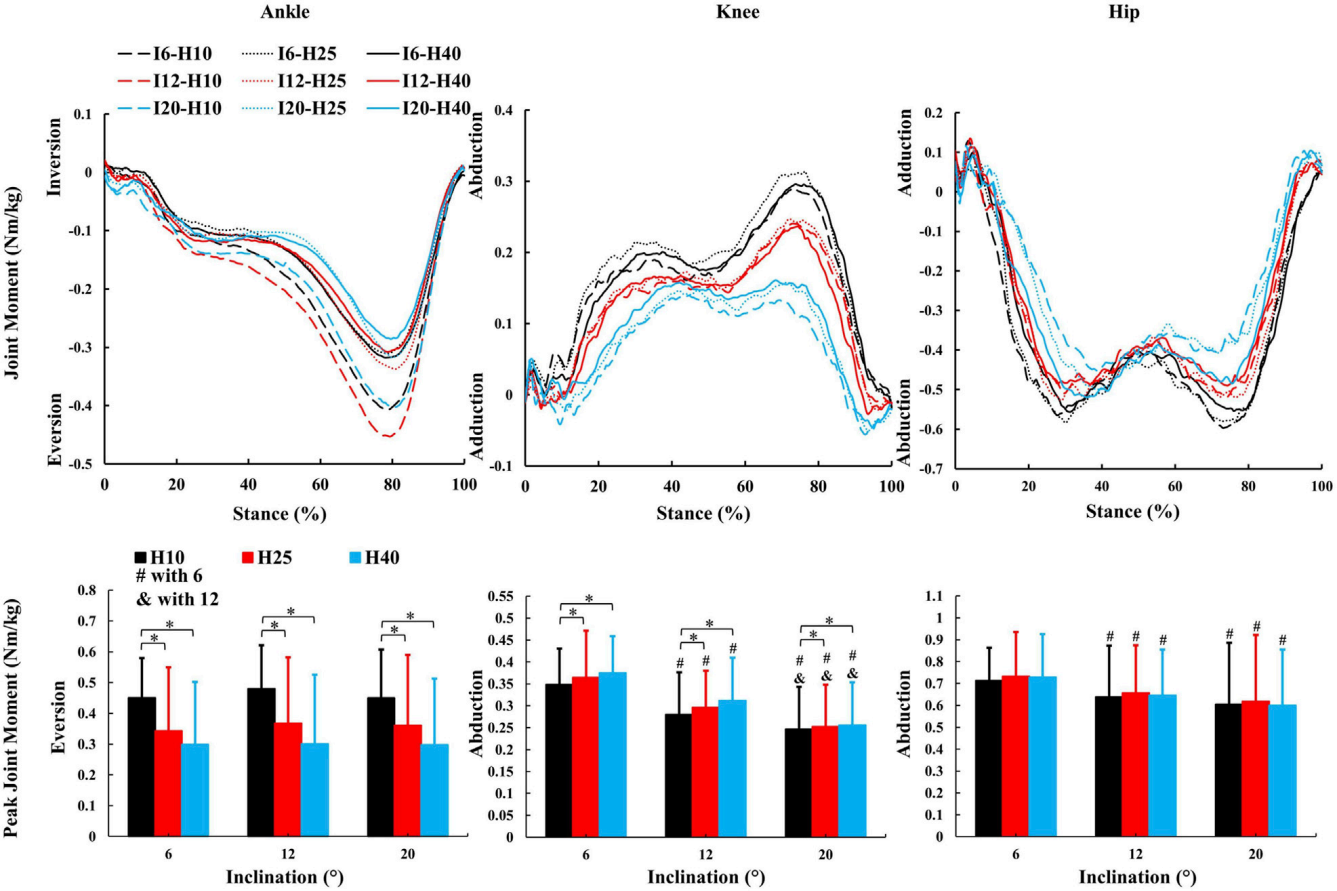
The average kinetic parameters (frontal joint moment and peak value) during the stance phase of uphill walking with various inclinations and heel-to-toe drops (I - inclination, H - HTD). *, #, & indicate a significant difference (*p* < 0.05).

**FIGURE 6:**
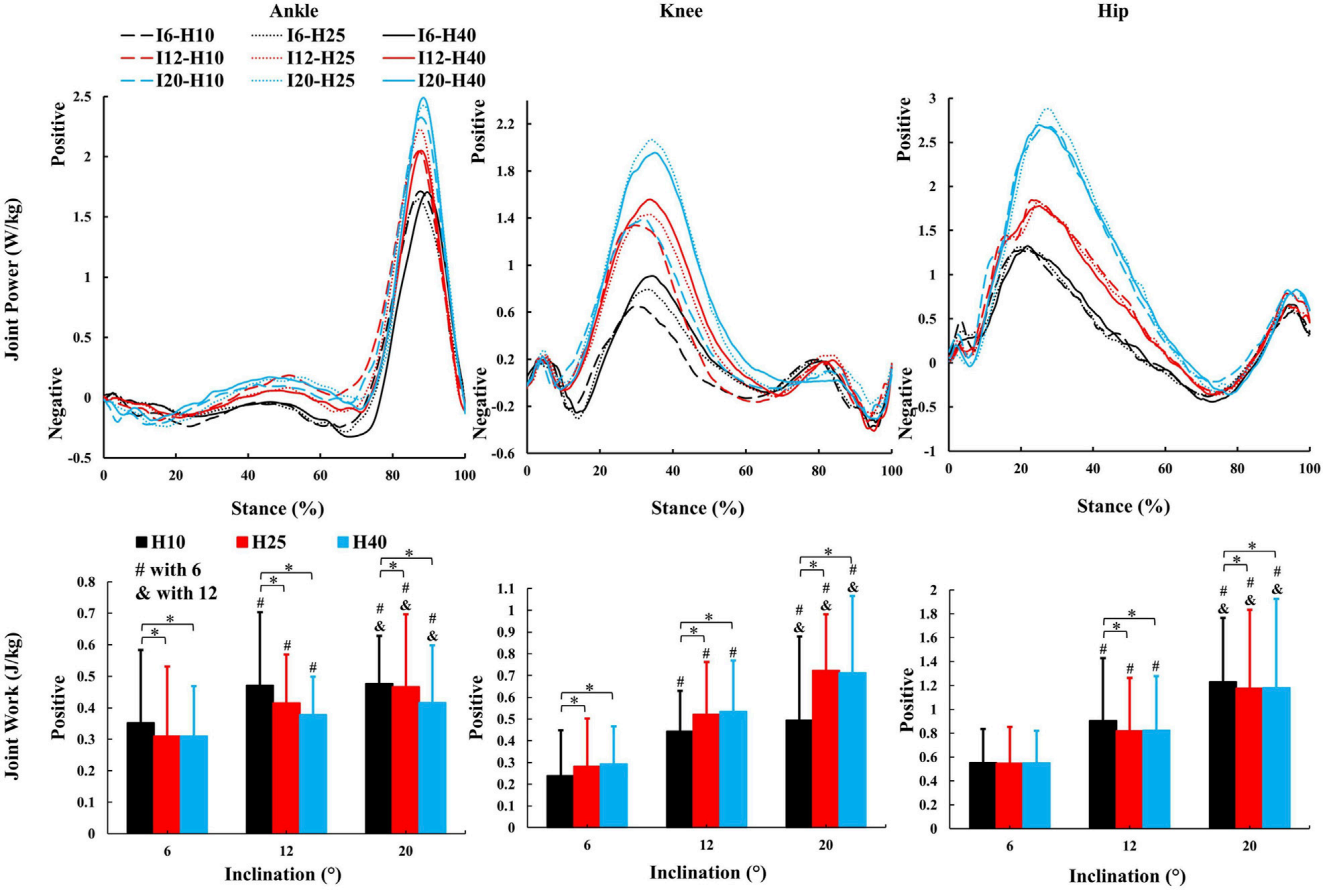
The average kinetic parameters (sagittal joint power and work) during the stance phase of uphill walking with various inclinations and heel-to-toe drops (I - inclination, H - HTD). *, #, & indicate a significant difference (*p* < 0.05).

No inclination ✕ HTD interaction effects were observed for kinetic parameters. A significant main effect of inclinations (Figures 4–6) was observed for peak ankle plantarflexion moment (F = 5.246, *p* = 0.033, $\eta_{p}^{2}$ = 0.37), ankle sagittal positive work (F = 17.04, *p* = 0.001, $\eta_{p}^{2}$ = 0.81), peak knee extension moment (F = 8.16, *p* = 0.011, $\eta_{p}^{2}$ = 0.49), peak knee abduction moment (F = 18.38, *p* < 0.001, $\eta_{p}^{2}$ = 0.82), knee sagittal positive work (F = 89.31, *p* < 0.001, $\eta_{p}^{2}$ = 0.91), peak hip extension moment (F = 58.74, *p* < 0.001, $\eta_{p}^{2}$ = 0.94), peak hip abduction moment (F = 7.76, *p* = 0.014, $\eta_{p}^{2}$ = 0.46), hip sagittal positive work (F = 23.67, *p* < 0.001, $\eta_{p}^{2}$ = 0.86). There was no significant main effect observed for inclination in remaining kinetic parameters.

A significant main effect of HTD levels (Figures 5, 6) was observed for peak ankle eversion moment (F = 8.18, *p* = 0.012, $\eta_{p}^{2}$ = 0.58), ankle sagittal positive work (F = 18.92, *p* < 0.001, $\eta_{p}^{2}$ = 0.68), peak knee extension moment (F = 22.56, *p* < 0.001, $\eta_{p}^{2}$ = 0.71), peak knee abduction moment (F = 4.3, *p* = 0.03, $\eta_{p}^{2}$ = 0.28), knee sagittal positive work (F = 16.42, *p* = 0.001, $\eta_{p}^{2}$ = 0.65), hip sagittal positive work (F = 4.45, *p* = 0.033, $\eta_{p}^{2}$ = 0.33). There was no significant main effect observed for HTD in remaining kinetic parameters.

### 3.4 Both inclination and HTD influence the muscle synergy parameters

**FIGURE 7:**
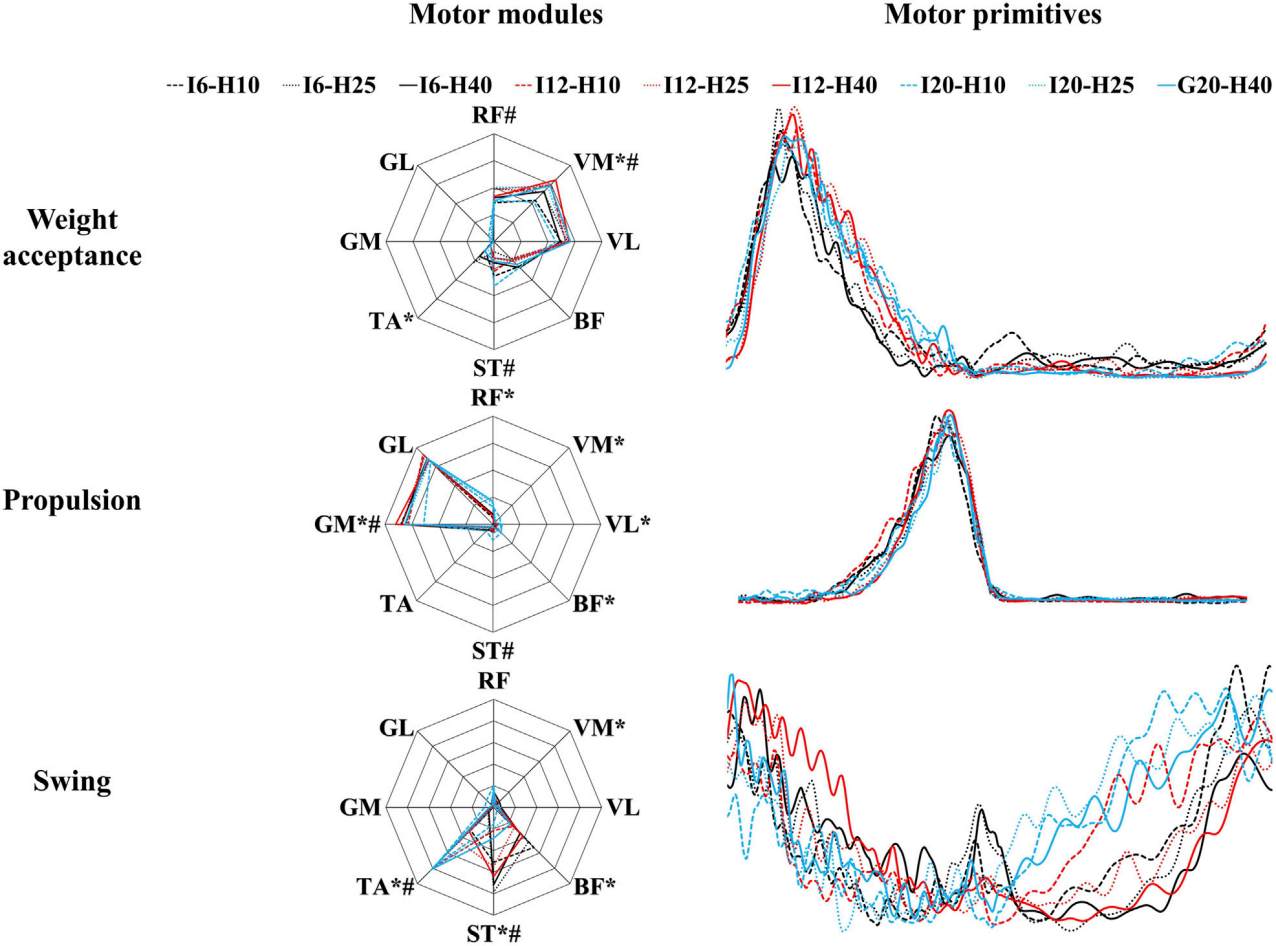
The motor modules and motor primitives during uphill walking with various inclinations and heel-to-toe drops (I - inclination, H - HTD). * indicates statistically significant differences (*p* < 0.05) among outcomes for varying inclines. # indicates statistically significant differences (*p* < 0.05) among outcomes for varying HTD.

No inclination ✕ HTD interaction effects were observed for muscle synergy parameters. There was no significant difference in the number of synergies across all inclination and HTD levels (Figure 7). Each synergy was associated with a different gait phase (weight acceptance, propulsion and swing) and ordered according to the CoA of each motor primitive (Table 2). A significant main effect of inclination levels (Table 2) was observed for CoA of weight acceptance (F = 16.1, *p* < 0.001, $\eta_{p}^{2}$ = 0.14), Swing (F = 3.48, *p* = 0.035, $\eta_{p}^{2}$ = 0.08) and FWHM in weight acceptance (F = 9.36, *p* < 0.001, $\eta_{p}^{2}$ = 0.09), Swing (F = 3.885, *p* = 0.024, $\eta_{p}^{2}$ = 0.09). There was no significant main effect observed for inclination in propulsion. A significant main effect of HTD level s (Table 2) was observed for CoA in propulsion (F = 6.32, *p* = 0.002, $\eta_{p}^{2}$ = 0.06). There was no significant main effect observed for HTD in weight acceptance and Swing.

**TABLE 2:** Mean (SD) CoA and FWHM of the motor primitives on three inclinations with three HTD-levels.

Variable					ANOVA results									
	HTD				Interaction			Inclination			HTD			Post-hoc
	Inclination	10 mm	25 mm	40 mm	*F*	*p*	$\eta_{p}^{2}$	*F*	*p*	$\eta_{p}^{2}$	*F*	*p*	$\eta_{p}^{2}$	
CoA [%]														
Weight acceptance	6°	13.85 (4.46)	12.02 (4.18)	13.01 (3.29)	2.22	.07	.04	16.1	<.01	.14	1.60	.2	.02	6° < 12°, 6° < 20°
	12°	14.39 (4.63)	16.86 (2.98)	16.65 (3.45)										
	20°	15.28 (3.8)	16.4 (4.12)	17.34 (3.1)										
Propulsion	6°	37.12 (2.7)	38.3 (2.96)	38.7 (3.87)	.13	.97	<.01	.77	.46	.01	6.32	**<.01**	.06	10 mm < 25mm, 10 mm < 40 mm
	12°	37.22 (3)	39.23 (2.68)	38.97 (2.45)										
	20°	37.67 (3.48)	39.18 (3.53)	39.14 (2.64)										
Swing	6°	91.59 (6.43)	94.46 (5.3)	86.02 (16.34)	1.87	.12	.08	3.48	.04	.08	.43	.65	.01	6° > 20°, 12° > 20°
	12°	87.32 (6.95)	92.87 (4.9)	93.52 (5.51)										
	20°	85.55 (8.28)	82.79 (10.71)	88.96 (8.33)										
FWHM														
Weight acceptance	6°	10.53 (4.02)	10.43 (4.25)	9.37 (3.07)	.43	.79	.01	9.36	<.01	.09	9.36	.38	.01	6° < 12°, 6° < 20°
	12°	13.23 (3.98)	13.78 (4.24)	12.8 (4.55)										
	20°	13.66 (5.38)	11.87 (6.01)	12.06 (4.39)										
Propulsion	6°	9.22 (4.08)	9 (3.61)	10.1 (3.67)	1.15	.33	.02	1.49	.23	.01	2.54	.08	.02	
	12°	10.9 (3.73)	8.74 (3.04)	9.02 (3.23)										
	20°	9.58 (2.8)	8.12 (3.11)	8.23 (3.19)										
Swing	6°	15.11 (8.48)	16.58 (5.86)	17.08 (5.5)	.22	.93	.01	3.89	.02	.09	.05	.96	<.01	12° < 20°
	12°	15.13 (8.28)	13.32 (8.44)	13.18 (1.76)										
	20°	19.43 (8.99)	21.62 (8.61)	21.18 (10.42)										

During the weight acceptance phase, a significant main effect of inclination levels (Figure 7) was observed for VM (F = 8.12, *p* < 0.001, $\eta_{p}^{2}$ = 0.08), TA (F = 10.72, *p* < 0.001, $\eta_{p}^{2}$ = 0.1). There was no significant main effect observed for inclination in remaining muscles. A significant main effect of HTD levels (Figure 7) was observed for RF (F = 3.95, *p* = 0.021, $\eta_{p}^{2}$ = 0.04), VM (F = 6.28, *p* = 0.002, $\eta_{p}^{2}$ = 0.06), ST (F = 13.13, *p* < 0.001, $\eta_{p}^{2}$ = 0.12). There was no significant main effect observed for HTD in remaining muscles.

During the propulsion phase, a significant main effect of inclination levels (Figure 7) was observed for RF (F = 26.63, *p* < 0.001, $\eta_{p}^{2}$ = 0.21), VM (F = 4.61, *p* = 0.011, $\eta_{p}^{2}$ = 0.04), VL (F = 4.06, *p* = 0.019, $\eta_{p}^{2}$ = 0.04), BF (F = 6.76, *p* = 0.001, $\eta_{p}^{2}$ = 0.06), GM (F = 4.372, *p* = 0.014, $\eta_{p}^{2}$ = 0.04). There was no significant main effect observed for inclination in remaining muscles. A significant main effect of HTD levels (Figure 7) was observed for ST (F = 5.23, *p* = 0.006, $\eta_{p}^{2}$ = 0.05), GM (F = 4.38, *p* = 0.014, $\eta_{p}^{2}$ = 0.04). There was no significant main effect observed for HTD in remaining muscles.

During the swing phase, a significant main effect of inclination levels (Figure 7) was observed for VM (F = 6.2, *p* = 0.003, $\eta_{p}^{2}$ = 0.08), BF (F = 17.28, *p* < 0.001, $\eta_{p}^{2}$ = 0.19), ST (F = 25.35, *p* < 0.001, $\eta_{p}^{2}$ = 0.26), TA (F = 53.79, *p* < 0.001, $\eta_{p}^{2}$ = 0.42). There was no significant main effect observed for inclination in remaining muscles. A significant main effect of HTD levels (Figure 7) was observed for ST (F = 10.26, *p* < 0.001, $\eta_{p}^{2}$ = 0.12), TA (F = 7.2, *p* = 0.001, $\eta_{p}^{2}$ = 0.09). There was no significant main effect observed for HTD in remaining muscles.

## 4 Discussion

This study investigates the impact of HTD on biomechanical and neuromuscular responses during uphill walking at various inclinations. The existing literature extensively explores the impact of HTD on level walking (Stefanyshyn et al., 2000; Cowley et al., 2009; Barkema et al., 2012; Cronin, 2014). Our study, however, extends this inquiry to the effects of HTD across different slope inclinations, which is an issue less comprehensively investigated. Furthermore, previous research identifies a significant shift in gait parameters beginning at a 6°–9° incline (Prentice et al., 2004; Lay et al., 2006), indicating that biomechanical adaptations to inclined walking distinctly diverge from those of level walking at this threshold. Consequently, we selected 6° as the initial inclination for our study to examine how HTD influences the biomechanical properties of the human body under uphill conditions. Our findings reveal that adjusting HTD not only influences lower limb kinematics, kinetics, and muscle synergy parameters but also reduces biomechanical strain in uphill conditions. This underscores the practical implications of HTD adjustments in enhancing locomotion strategies during uphill walking.

An increase in inclinations during uphill walking demands more force and energy from the human body, making it a more challenging activity. Our study finds a decrease in gait speed and step length, and an increase in the stance and double support phases for enhanced stability (Table 1), coincides with other studies (Kimel-Naor et al., 2017; Sarvestan et al., 2021; Strutzenberger et al., 2022). Additionally, inclines lead to increased sagittal plane angles at the hip, knee, and ankle joints during uphill walking (Figure 3), coincides with other studies (Lay et al., 2006; Sarvestan et al., 2021). This requires the joints to exert more force to support body weight and reduces the range of motion, thus increasing the burden on the joints and necessitating stronger muscle strength. Our study finds that HTD does not significantly impact temporo-spatial and kinematic parameters during uphill walking (Table 1), with the exception of the range of motion at the ankle joint (Figure 3). Unlike on flat ground, where HTD can influence gait parameters and joint angles (Menant et al., 2009; Mika et al., 2012; Cronin, 2014), the body’s focus during uphill walking shifts towards maintaining balance and stability against the slope’s increased challenge (Hong et al., 2015; Alexander and Schwameder, 2016), diminishing the significance of HTD variation on posture. Moreover, the range of HTD variation examined in this study may not have been sufficient to significantly affect these parameters, suggesting that during uphill walking, the biomechanical challenges posed by the slope might overshadow the effects of HTD adjustments.

Changes in the joint moment on the frontal plane of the ankle, knee, and hip joints can affect the distribution of load across muscles and joints during walking (Barkema et al., 2012; Simonsen et al., 2012; Wen et al., 2019). Higher HTD lessens ankle eversion and its moment on slopes (Figure 5), potentially reducing ankle injury risk, especially in individuals prone to sprains. Interestingly, this finding is inconsistent with the results of studies conducted on level ground walking (Barkema et al., 2012). Increased HTD may shift gait from heel-strike to midfoot or forefoot strike during uphill walking (Vernillo et al., 2017), decreasing ankle eversion moments compared to level ground walking (Yu et al., 2022). A reduced knee abduction moment during uphill walking suggests less lateral knee stability is needed (Wen et al., 2019). However, a higher moment with increased HTD indicates greater stress on the knee’s lateral structures, such as the meniscus and collateral ligaments (McWilliams et al., 2014).

In this study, as the inclination increased, there was an increase in positive work in the sagittal plane at the hip, knee, and ankle joints (Figure 6), which coincides with the results of previous studies (Alexander et al., 2017; Yang et al., 2019), indicating that these joints need to generate more force and energy to overcome gravity and the incline during uphill walking. The most notable observation from the study is that an increase in HTD led to a reduction in positive work in the sagittal plane at the ankle and hip joints during uphill walking, concomitantly with an elevation in positive work at the knee joint. Increased HTD may limit ankle dorsiflexion and alter body posture, reducing positive work at the ankle and hip joints. Consequently, the knee joint may compensate with increased positive work to preserve gait efficiency.

Several studies have demonstrated that an increase in inclination appears to have minimal impact on most patterns of muscle synergy during walking (Rozumalski et al., 2017; Dewolf et al., 2020). We also found that HTD does not significantly affect the number of muscle synergy patterns during uphill walking (Figure 7), which showed that the overall neuromuscular control strategies tend to remain consistent with varied inclinations and HTD during uphill walking. However, steeper inclination or increased HTD may lead to change in the activation level and duration of certain muscle, aligning with the changing demands of joint dynamics (Saito et al., 2018).

During the weight acceptance phase of uphill walking, an increase in inclination was associated with higher CoA and FWHM values (Table 2), suggesting later and more prolonged muscle activation. This shift in muscle activation towards propulsion and prolonged engagement for uphill stability may stem from the increased force and stability requirements of lower limb muscles to counteract gravity and facilitate ascent (Alexander and Schwameder, 2016). At the same time, the increased HTD heightens the activation of the vastus medialis during uphill walking, while the activation of the semitendinosus is diminished (Figure 7). This finding is consistent with studies conducted on level ground walking (Simonsen et al., 2012). This result may be explained by the fact that increased activation of the vastus medialis helps to stabilize the pelvis and knee joint, as well as to absorb shock. The reduced activation of the semitendinosus may be attributable to changes in the foot strike pattern (decreased ankle plantarflexion) and the reduced degree of knee flexion caused by the incline.

During the propulsion phase, as the inclination increases, there is an augmented activation of the rectus femoris, vastus medialis, vastus lateralis, and biceps femoris (Figure 7). This finding is consistent with previous research (Franz and Kram, 2012). Uphill walking increases the demand on the quadriceps for knee stabilization and propulsion due to the added gravitational force (Zai and Grabowski, 2020). The rectus femoris and biceps femoris are particularly important for generating the required vertical propulsive forces through knee extension (Haggerty et al., 2014). Additionally, the vastus medialis and lateralis contribute to knee stability, preventing deviations and promoting efficient, safe gait (Wen et al., 2019). An increase in HTD results in a higher CoA (Table 2), suggesting that an augmented HTD may influence the mechanical state of the foot and the activation patterns of the musculature. To uphill walk effectively, the musculature of the lower limbs must work in a more coordinated fashion to generate enough propulsive force.

During the swing phase, as the inclination increases, there is an elevation in the activation level of the tibialis anterior muscle (Figure 7). A possible explanation for this might be that the ankle joint may require a greater degree of dorsiflexion during the swing phase to prepare for the subsequent foot strike (Sarvestan et al., 2021). The increase in inclination also results in a reduced CoA, accompanied by an increase in the FWHM (Table 2). Ascending inclines may require earlier and prolonged muscle activation to meet the demands of limb clearance and forward propulsion in uphill walking.

It should be acknowledged that the results of this study, only derived from young and healthy male participants, may not be generalized to other populations with varying ages, genders, or health conditions. Furthermore, the analysis of muscle synergies was limited lower limb muscles, which are recognized as primary contributors during uphill walking (Pickle et al., 2016). However, it is important to acknowledge that the muscles of the trunk, which were not included in our analysis, may also play a compensatory role during uphill walking (Li et al., 2022; Yamato et al., 2023). This highlights a potential area for future research to explore the role of muscle compensation in different populations and under various walking conditions.

Adjusting the HTD through various insoles or shoes with adjustable features is crucial for enhancing biomechanical and neuromuscular performance, especially in slope walking. This adjustment can prevent falls and improve muscle training. In practical applications, specialized footwear with adjustable HTD is tailored for different terrains, such as shoes with higher drops that offer additional cushioning and support during uphill movements, helping to prevent overuse injuries and enhance stability. This research area promises significant potential for future studies, focusing on developing footwear that can adapt to diverse environmental conditions to maximize safety and physical performance.

## 5 Conclusion

This study demonstrates that an increase in the inclination leads to changes in biomechanical and neuromuscular responses during uphill walking and the adjustment of HTD can modulate these responses during uphill walking. However, the present study suggests that an increased HTD may lead to elevated loads on the knee joint and these adverse effects need more attention.

## Data Availability

The original contributions presented in the study are included in the article/Supplementary material, further inquiries can be directed to the corresponding authors.
